# Man Complaining of Sore Throat

**DOI:** 10.1016/j.acepjo.2025.100178

**Published:** 2025-05-22

**Authors:** Jasmine Yu, Payton Sullivan, Jasmine Thompson, Joseph S. Colla

**Affiliations:** 1 Department of Emergency Medicine, University of Illinois Hospital and Health Sciences System, Chicago, Illinois, USA; 2 Miami University, Oxford, Ohio, USA

**Keywords:** esophageal perforation, foreign body ingestion, ultrasound, subcutaneous air

## 1 Patient Presentation

**Figure 1:**
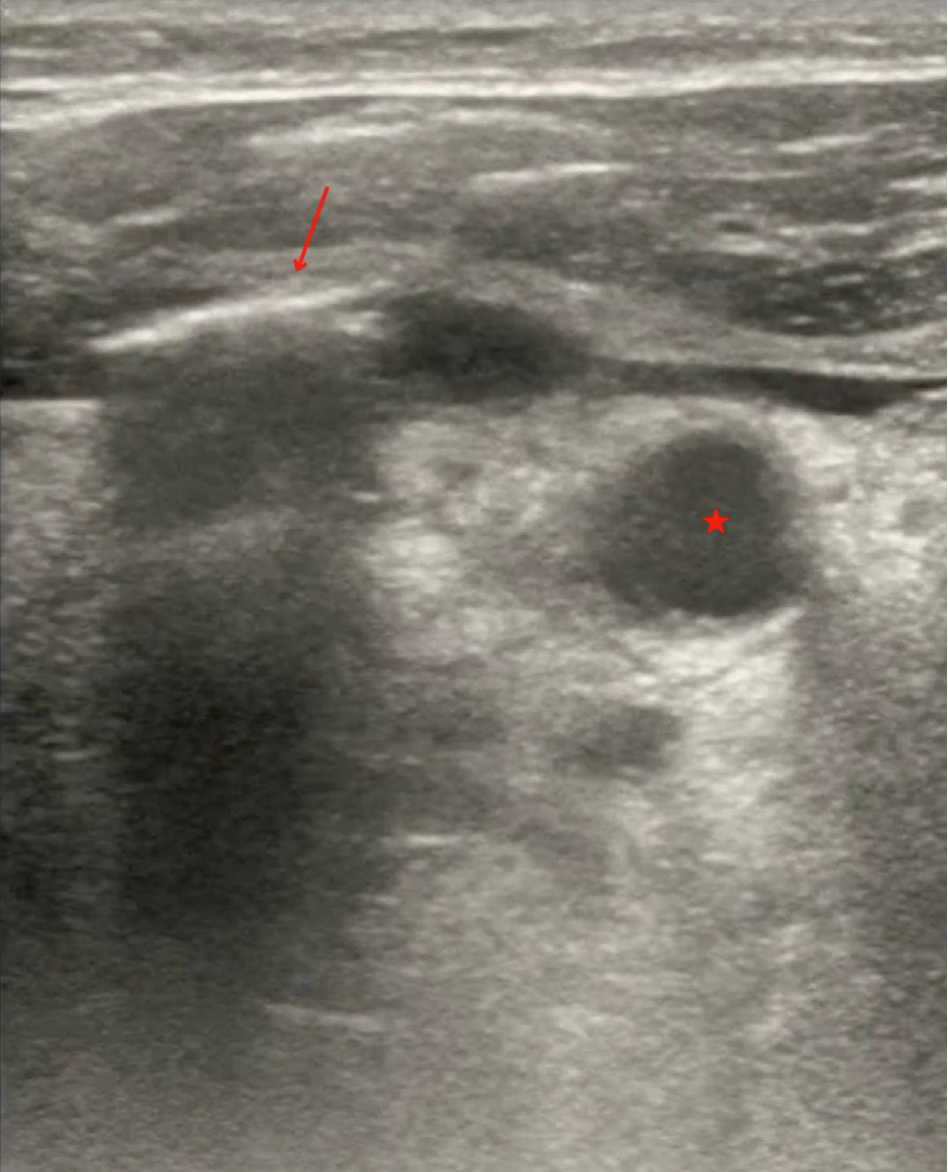
Bedside ultrasound showed shadowing under an area of subcutaneous air (arrow) adjacent to the trachea (star).

**Figure 2:**
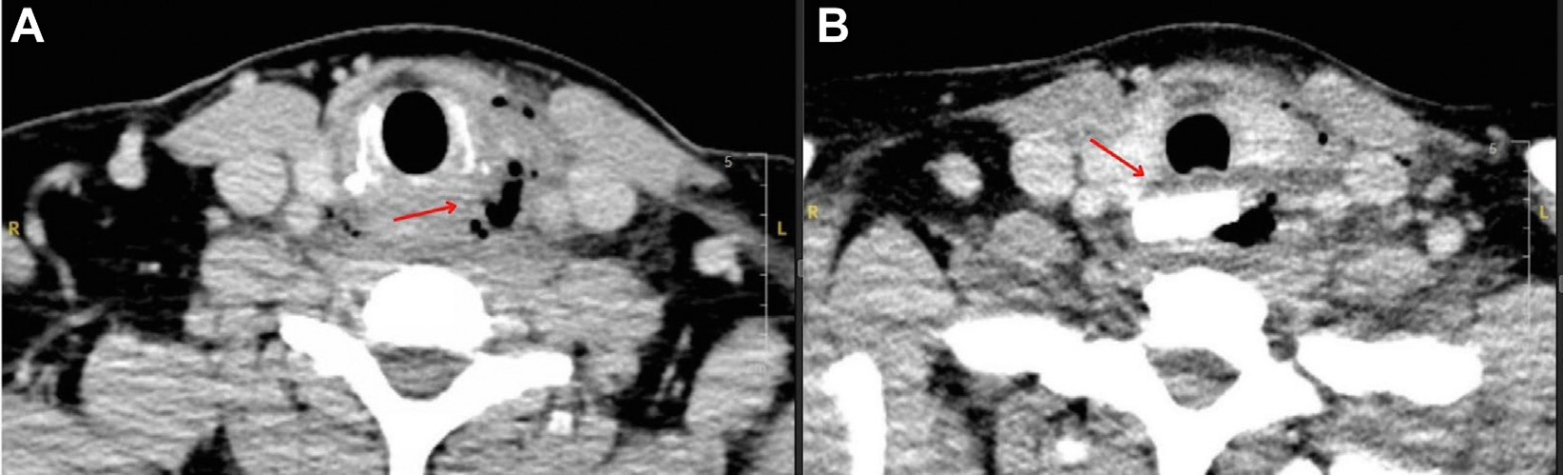
Computed tomography of the neck shows cross-sections demonstrating (A) anterior subcutaneous gas correlating with ultrasound image (Fig 1) and (B) rectangular metallic density.

**Figure 3:**
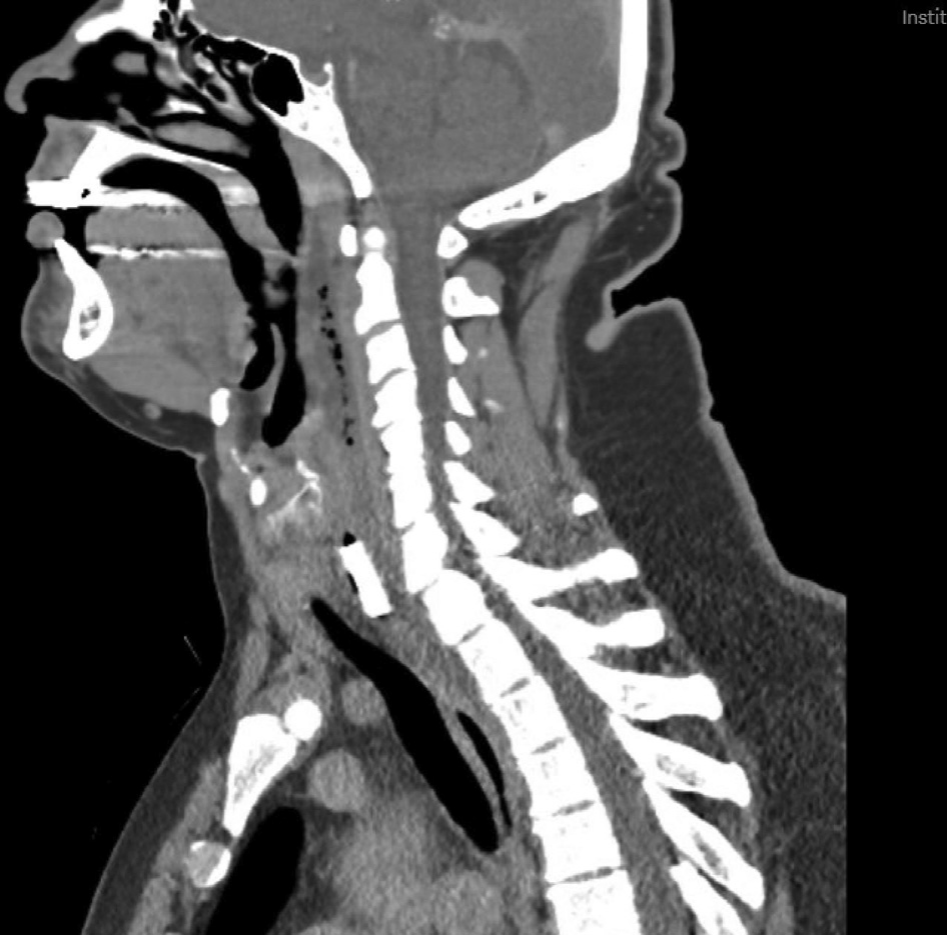
Computed tomography of the neck demonstrated a rectangular metallic density with multiple foci of gas consistent with esophageal perforation.

A 44-year-old man presented to the emergency department with a sore throat, fever, and concerns that he may have swallowed glass. He stated that the day prior he was eating in an area where broken glass was present. Since then, he experienced pain with odynophagia and dysphagia. Upon arrival, he was tachycardic, hypotensive (with a blood pressure of 98/57 mm Hg), and febrile, with a temperature of 38.5 °C. A bedside ultrasound revealed subcutaneous air adjacent to the trachea (Fig 1 and Video). A neck computed tomography scan with contrast was arranged for further evaluation (Figs 2 and 3).

**Video:**
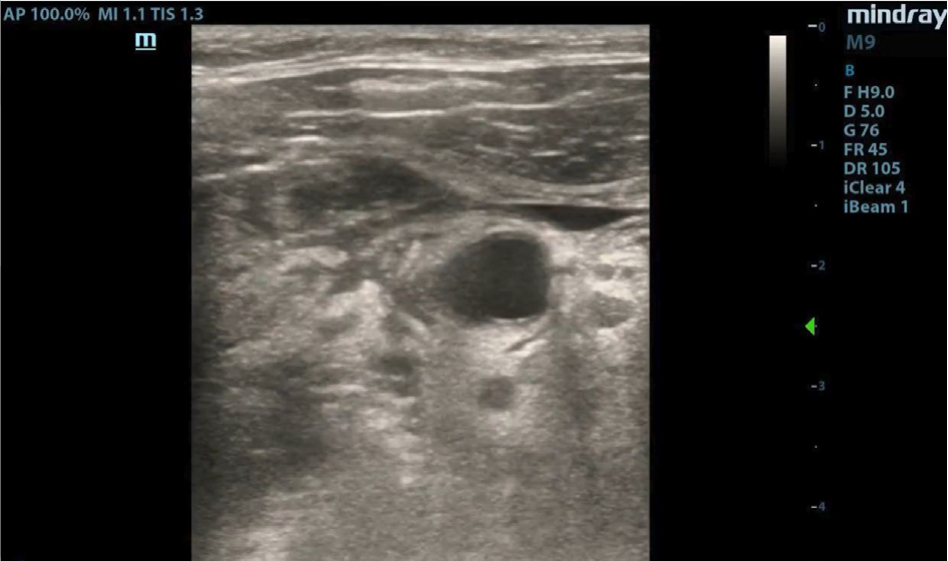
Ultrasound revealing subcutaneous air adjacent to the trachea.

## 2 Diagnosis: Esophageal Foreign Body with Esophageal Perforation

The patient underwent a rigid esophagoscopy. The operative note indicated that there was a 3 × 3-cm piece of glass located just below the upper esophageal sphincter, surrounded by inflammation and purulence with a large perforation at the left lateral wall of the esophagus. An external neck exploration was formed without obvious abscess or perforation. The perforation appeared to be contained within a layer of fibrinous tissue. The patient was fluid resuscitated, placed on broad-spectrum antibiotics, and started on tube feeds. A gastrografin esophagram was performed 8 days later was negative for esophageal leak or stricture.

Esophageal perforation is a serious, life-threatening medical emergency. X-rays are an easy but insensitive screening test. Contrast esophagography is the preferred diagnostic test for confirming esophageal perforation; however, computed tomography scans are more sensitive for detecting foreign bodies.^1^^,^^2^ Ultrasound has also become a popular diagnostic tool in cases of free air and is notable for detecting soft tissue air with a sensitivity of 100%.^3^, ^4^, ^5^

## Funding and Support

By *JACEP* Open policy, all authors are required to disclose any and all commercial, financial, and other relationships in any way related to the subject of this article as per ICMJE conflict of interest guidelines (see www.icmje.org). The authors have stated that no such relationships exist.

## Conflict of Interest

All authors have affirmed they have no conflicts of interest to declare.
